# Good handling practice of parenterally administered medicines in neonatal intensive care units – position paper of an interdisciplinary working group

**DOI:** 10.3205/dgkh000436

**Published:** 2023-05-03

**Authors:** Irene Krämer, Rangmar Goelz, Christian Gille, Christoph Härtel, Rachel Müller, Thorsten Orlikowsky, Brar Piening, Sebastian Schubert, Arne Simon, Katharina Wolf, Bianka Rösner, Martin Exner

**Affiliations:** 1 Department of Pharmacy, University Medical Center, Johannes Gutenberg University Mainz, Mainz, Germany; 2 Department of Neonatology, Tübingen University Children's Hospital, Tübingen, Germany; 3 Clinic for Neonatology, University Hospital for Paediatrics and Adolescent Medicine Heidelberg, Heidelberg, Germany; 4 University Hospital Würzburg, Department of Pediatrics, Würzburg, Germany; 5 Pharmacy of Saarland University Hospital, Homburg, Germany; 6 Section of Neonatology and Paediatric Intensive Care Medicine, University Hospital Aachen, Aachen, Germany; 7 Institute for Hygiene and Environmental Medicine, Charité – Universitätsmedizin Berlin, Berlin, Germany; 8 Special interest group for Paediatric Pharmacy, German Society of Hospital Pharmacists (ADKA e.V.), Germany; 9 Paediatric Oncology and Haematology, Children's Hospital, Saarland University Hospital, Homburg, Germany; 10 German Society for Paediatric Infectiology, Berlin, Germany; 11 University Pharmacy, Tübingen University Hospital, Tübingen, Germany; 12 Charité – Universitätsmedizin Berlin, Centre for Gynaecology, Paediatrics and Adolescent Medicine, Clinic for Neonatology, Specialist Paediatric Intensive Care Nurse, Berlin, Germany; 13 Prevention and Outbreak Management/One health at the Institute of Hygiene and Public Health, WHO CC University Hospital Bonn for the Board of the German Society for Hospital Hygiene (DGKH), Bonn, Germany

**Keywords:** prescription, preparation, reconstitution, administration, parenterally administered medicines, good handling practice, neonatal intensive care, bloodstream infection

## Abstract

This position paper, developed by an interdisciplinary expert group of neonatologists, paediatric infectious disease physicians, clinical pharmacists and specialists for the prevention and control of nosocomial infections, describes the “Good handling practice of medicines parenterally administered to patients on NICUs”. It takes equal account of patient safety and the specialties of neonatal intensive care regarding feasibility and proportionality. The overall concept is perceived as a “learning system”, in which open communication within the health-care team relating to medication errors and critical incidents enables continuous development and improvement to ensure patient safety. In our opinion, pharmacists, who are responsible for the supply of ready-to-administer parenteral medicinal products for neonatal intensive care patients, as well as the hygiene staff responsible on site are integral parts of the interdisciplinary treatment team. Risks of the current clinical practice of parenteral treatment of NICU patients are discussed in detail and recommendations for safety-relevant procedures are given.

## Abstract (de)

Das hier vorgelegte Positionspapier wurde von einer interdisziplinären Expertengruppe, bestehend aus Neonatologen, pädiatrischen Infektiologen, klinischen Pharmazeuten und Spezialisten für Prävention und Kontrolle nosokomialer Infektionen, erarbeitet und beschreibt die „Gute Praxis der parenteralen Arzneimitteltherapie“. Diese trägt gleichermaßen der Sicherheit der Patienten und den besonderen Gegebenheiten einer neonatologischen Intensivstation Rechnung, wobei auch Machbarkeit und Verhältnismäßigkeit der praktischen Umsetzung berücksichtigt werden. Das Gesamtkonzept wird als ein „lernendes System“ aufgefasst; das heißt Fehler (critical incidents) hinsichtlich der Patientensicherheit werden im Behandlungsteam offen kommuniziert, sodass eine stetige Weiterentwicklung und Verbesserung der Abläufe möglich wird. Apotheker, die für die Versorgung von neonatologischen Intensivpatienten mit parenteral zu verabreichenden Arzneimitteln verantwortlich sind, gelten nach unserem Verständnis (genau wie das vor Ort zuständige Hygienefachpersonal) als integraler Bestandteil des interdisziplinären Behandlungsteams. Die Risiken, die mit der etablierten klinischen Praxis der parenteralen Therapie von Früh-/Reifgeborenen auf Intensivstationen einhergehen, werden von der Expertengruppe im Einzelnen dargestellt und es werden Hinweise für sicherheitsrelevante Vorgehensweisen gegeben.

## Background

Parenterally, mainly intravenously, administered medicines are an integral part of intensive care treatment for premature and mature but ill neonates. Parenteral drug therapy comprises the administration of medicinal products as injections, short infusions (SI) or continuous infusions (CI), and the administration of parenteral nutrition admixtures. Using parenteral medicines safely is a complex process and a challenge for all health-care professionals involved [1]. Five predominant aspects have to be considered:

- Safe prescription, preparation and administration of parenteral medicines are medical necessities at any given time (24/7) in a three-shift system at a neonatal intensive care unit (NICU).
- Reconstitution of licensed medicinal products should be carried out according to the information given in the summary of product characteristics (compare §4 (31) German Medicines Act (AMG)).
- Preparation of medicinal products by/under the responsibility of the prescribing physician must be carried out in accordance with §13 (2b) AMG and §4 (14) AMG.
- Hospital pharmacies executing aseptic preparation of ready-to-administer parenterals are not available 24/7 in almost all German hospitals running NICUs.
- Due to the federal character of the health-care system in Germany, quality and safety assurance requirements regarding the reconstitution and preparation of medicinal products in health-care establishments are hardly uniform.

For the development of recommendations for safety-relevant procedures, a working group (WG) was initiated by the department of neonatology at the University Hospital in Tübingen (RG, CG) and constituted in spring 2021. Neonatal intensive-care physicians (RG, CG, CH, TO), pediatric infectious disease specialists (AS, CH), clinical pharmacy specialists (KW, RM, SSCH, IK), a specialized neonatal intensive-care nurse (BR), and experts in hospital hygiene (BP, ME) are members of the WG and affiliated to university hospitals with Level 1 NICUs. RG and BR are mandated by the GNPI (Society for Neonatology and Pediatric Intensive Care Medicine), SSch represents the Special Interest group for Pediatric Pharmacy of the German Society of Hospital Pharmacists (ADKA e.V.) and AS is the mandated representative of the DGPI (German Society for Pediatric Infectious Disease), appointed member of the Commission for Hospital Hygiene and Infection Prevention (KRINKO) at the Robert Koch Institute since 2004 and coordinator of the KRINKO recommendations on neonatal intensive care. The coordination and specific literature search were carried out by RM (Pharmacy of the University Hospital in Homburg, TELEKASPER project). IK, CG and AS were members of the KRINKO BfArM (Federal Institute for Medicinal Products and Medical Devices) working group [2]. All members of the WG were actively involved in the development of this manuscript, which was submitted to the board of the DGKH (German Association for Hospital Hygiene) for critical evaluation prior to publication.

## Objectives of the position paper

This position paper describes the good handling practice of medicines administered parenterally to patients on NICUs’. It takes equal account of patient safety and the specialties of neonatal intensive care regarding feasibility and proportionality. The overall concept is perceived as a “learning system”, in which open communication within the health-care team relating to medication errors and critical incidents [3] enables continuous development and improvement to ensure patient safety [4], [5], [6]. In our opinion, pharmacists who are responsible for the supply of ready-to-administer parenteral medicinal products for neonatal intensive care patients and the hygiene staff responsible on site are integral parts of the interdisciplinary treatment team.

Risks of the current clinical practice of parenteral treatment of NICU patients are discussed in detail and recommendations for safety-relevant procedures are given. The risks associated with the manual handling of parenteral therapy is always higher than those associated with standardized and automated processes, because humans are involved at various steps of the procedures and a residual probability of error exists [7]. Thus, constant vigilance of each team member should be induced to ensure the best treatment quality and patient safety in everyday clinical practice. For personnel, structural, organizational, and functional reasons, daily practice on site may deviate from given recommendations and standards. However, this should not entail a higher risk for patients.

## Good handling practice of parenterally administered medicines on a NICU

Essential steps and special features of safe parenteral drug therapy for preterm and sick, term infants on the NICU are listed below:

- Specified dosing of medicines for premature infants in accordance with gestational age, birth weight, organ function, and chronological age [8],
- reconstitution according to the summary of product characteristics of licensed products,
- preparation of complex, patient-specific infusion solutions [4], [9], [10], [11], [12],
- proper storage of infusion solutions prior to administration [10],
- safe administration to the patient [13],
- quality assurance, e.g. by assurance of process quality (internal audits by specialists) and measurement of outcome quality, e.g. vascular catheter-associated infection rates according to NEO KISS, a surveillance system for nosocomial infections [14], or by voluntary reporting systems for critical incidents or near misses as described by Snijders et al. [3],
- education (imparting theoretical knowledge) and training (imparting practical skills) of the health care team [10], [15], [16].

The WG recommends writing the critical processes in internal standard operating procedures (SOP) for prescription, preparation and administration. The SOPs should encompass the ward-specific formulary and the specific **dosing guidance for NICU patients**. The SOPs should be approved by a specialist team of nursing, medical staff, pharmacists, and hospital hygienists.

The SOPs should be developed on the basis of a risk analysis representing the specific circumstances and processes of the individual NICU. The risk analysis serves to initiate structured considerations for the implementation of concrete measures for risk minimization [6], [17]. This can also include decisions for mandatory preparation of particular ready-to-administer parenterals in a clean room of the pharmacy instead of preparing them on the ward [10].

## Interpretation guide for the inspection of preparing sterile medicinal products according to § 13 (2b) AMG in health-care establishments

The “Interpretation guide for the inspection of preparing sterile medicinal products” according to § 13 (2b) AMG is addressed to the German federal inspection authorities and describes how pharmaceutical standards are to be followed by physicians when preparing medicinal products for individual patients in health-care establishments. The interpretation guide was originally developed as an official guideline by the working group on medicinal products, pharmacies, transfusions and narcotics of the German federal health authorities. Despite considerable objections from medical [18] and pharmaceutical societies, it was adopted in 2018 as a recommendation [19]. The critical questioning of essential aspects of the document presented by the KRINKO was not regarded.

It is stated in the introduction that the document should be respected by physicians, thus also by neonatologists and pediatric intensive-care physicians. The German Medical Association (BÄK) and the Drug Committee of the German Medical Association (AkdÄ) [20] stated:

*“The primary objective of such a guideline should be to improve the safe use of medicines. The BÄK and the AkdÄ consider that it is an indispensable prerequi* *site* *for the successful implementation of such recommendations in medical practice that these are sen* *sible, practicable and implementable requirements in order to support the assurance of quality and safety of the treatment as well as the safety of the medicinal products administered. The requirements established must not restrict the freedom of medical care. Furthermore, such a guideline must not interfere with the regulatory competence of other bodies.”*

The members of our WG and other experts doubt whether the “vulnerability” of a patient group (e.g., adult vs. pediatric patients, normal ward vs. intensive care unit, oncology, neonatology) should play a role for reconstitution and preparation of parenterally administered medicinal products. The requirements must apply to any parenteral use of medicinal products *(“The strict demands on sterility must be applied to each parenteral preparation” *and* “The BÄK and the AkdÄ explicitly point out that the assessment of the vulnerability of an individual patient basically falls within the scope of medical responsibility and cannot be assessed by regulatory bodies” *[20]).

## Prescribing of parenterally administered medicines for NICU patients

There is no doubt that **electronic prescribing systems** are superior to manual (handwritten) prescribing on the NICU. Studies show a decrease in prescription errors after the introduction of e-prescribing [17], [21], [22], [23]. The prescribing process involves a particularly high risk of medication errors [24], [25], [26], [27]. In a recent meta-analysis, 4 to 35 medication errors per 1,000 patient days were reported, most of them related to dosage errors. Anti-infectives were incorrectly prescribed most frequently [26]. Manual prescribing in paper records, must be clearly legible and unambiguous (e.g. milligrams, micrograms instead of mg, µg). Hence, electronic prescriptions are clearly preferable.

The electronic prescribing system for neonatal intensive care patients should

- be integrated into the clinical information system (KIS),
- consider enteral nutrition when calculating parenteral nutrition,
- consider patient specific parameters relevant for dosing (e.g., gestational age, day of life, weight, percentiles, excretion, current laboratory values, etc.).

In addition, the prescribing software should include an automated plausibility check based on lower and upper dosage limits (per kg body weight) and generate warnings with regard to incompatibilities, osmolarity limits and drug interactions. Summaries of product characteristics and specific dosage recommendations for neonates should be available online at the same IT workstation.

**Figure 1:**
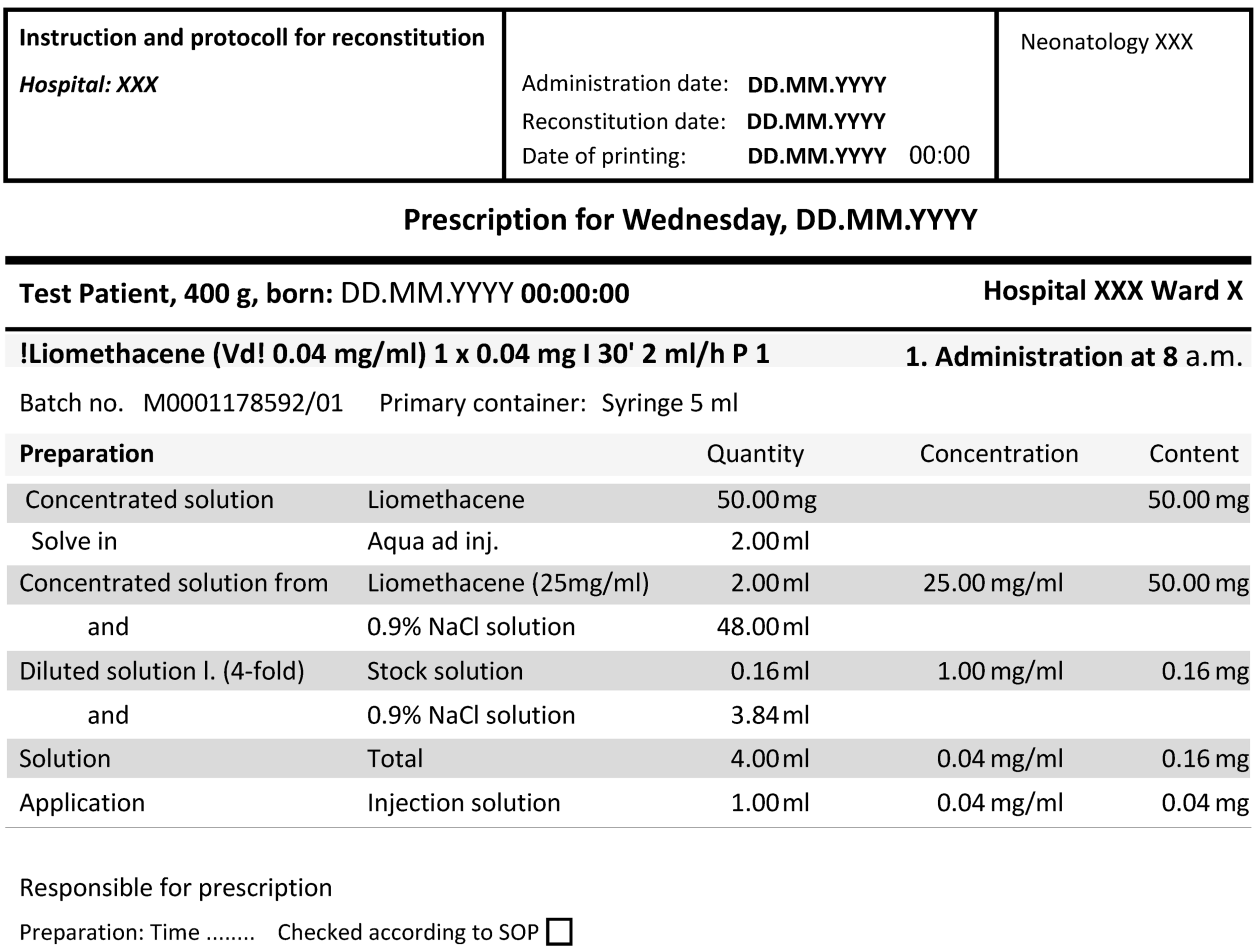
Exemplary prescription and working instruction for Liomethacene injection solution including surplus quantity for the dead volume in the administration device (source: software program, used in a NICU of a German university hospital)

The electronic prescriptions of parenterally administered medicines should be supplemented by clear instructions for the preparation of infusions ("detailed instructions for reconstitution"). Apart from handwritten initials and time points of preparation and administration, there should be as little handwritten information as possible. This ensures a detailed follow-up. Table 1 (Tab. 1) summarizes the most important steps in the prescription of parenteral medicines. A good example taken from the software program used on the NICU of a German University Hospital is shown in Figure 1 (Fig. 1). It illustrates the safe prescription and instructions for reconstitution and administration of Liomethacene.

Ideally, the prescription is authorized electronically. If the preparation (e.g., of parenteral nutrition admixtures) takes place in the pharmacy, an integrated prescribing and preparation software is preferred. The hard copy of complex prescriptions and their analogue transmission (via fax) followed by re-entry into a pharmacy’s preparation software creates avoidable medication errors and considerable efforts in additional checking.

The instructions for reconstitution/preparation should contain comprehensive information, including details of the devices to be used (specification of the line, syringe etc.). The hard copy should include the name of the prescribing physician, date and time of prescribing and printing. In case of ambiguities or deviations from the internal standard or specific clinical situations (e.g., excretion disorder, lack of suitable intravenous access), consultation with the prescribing physician or the senior physician in charge must be possible at any time (internal surveillance). The infusion instructions must be archived as part of the patient’s file.

When prescribing parenteral medicines, the **four-eye principle** is an important quality assurance measure to avoid medication errors. The physician who checks the prescription should be designated in advance [28]. In case of ambiguities, consultation with a senior neonatologist should always be possible. Preparation in the pharmacy requires a plausibility check of the prescription by the pharmacist according to §35 of the German Pharmacy Ordinance (*Apothekenbetriebsordnung*) [29].

Nowadays, electronic prescription software must be certified as medical device. Unfortunately, the availability of certified software is still very limited. There is an urgent need to develop suitable and certified software systems for the specific need of neonatal intensive care patients interfacing with other parts of the electronic patient record.

## Definitions and regulatory aspects of preparation of parenterally administered medicines for NICU patients

### Reconstitution

This is defined as manipulation to enable the use or application of a medicinal product with a marketing authorization in accordance with the instructions given in the summary of product characteristics or the patient information leaflet [18]. The authorized medicinal product may be marketed in the ready-to-use form (infusion solution in a bottle/bag, pre-filled syringe), as a concentrate, or as a powder. Powders or concentrates are dissolved and/or diluted with the solvent or vehicle solution given in the summary of product characteristics and thereby made ready to be administered to the patient. These operations are carried out by qualified personnel **immediately prior to use** and are referred to as reconstitution [30], [31].

According to the understanding of this WG, it is also a reconstitution if several consecutive dilution steps are required and the manufacturer’s product information does not contain any special information on the use in preterm and term neonates treated in the intensive care unit. For numerous medicinal products routinely used on NICUs for decades, detailed information for reconstitution is missing in the summary of product characteristics, although it is a prerequisite to ensure compliance with marketing authorization. Due to the very small amounts of active substance to be administered (and thus very small volumes of concentrates to be withdrawn), a sequential dilution of the stock solution is often necessary to ensure accurate dosing. According to the unanimous opinion of the WG, any manipulation to enable the administration of a single medicinal product is always a reconstitution and not categorized as preparation of medicinal products by physicians as defined in § 13 (2b) AMG (see below). According to AMG §13(1a), reconstitution of medicinal products by physicians in health-care establishments is not obliged to notify to the health care authorities. The competent supervisory authority ZLG (Central Authority of the Federal States for Health Protection regarding Medicinal Products and Medical Devices) states that it is irrelevant whether the solvent for reconstitution is enclosed with the marketed medicinal product or not [32].

### Preparation of parenterally administered medicines by or under the responsibility of the prescribing physician

The preparation of medicinal products without a manufacturing license includes the preparation of medicinal products under direct professional responsibility of a physician for personal use in a specific patient. When using licensed medicinal products as starting material, “preparation” means all manipulations that go beyond reconstitution and are not described in the summary of product characteristics. A typical example in NICUs is the preparation of macro- and micronutrient admixtures for parenteral nutrition. According to §67 AMG, the competent supervisory authority of the German federal states must be notified if medicinal products are prepared in clinical areas. Suitable forms are provided.

The responsible physician may delegate tasks to competent personnel in the clinical area. The extent of the assistance is primarily determined by the qualification and competence of the assisting personnel [33]. In practice, the preparation of parenteral medicinal products in the NICU is teamwork which involves different staff members taking over different tasks (delegation principle). Writing prescriptions is assigned to a certain physician, but the prescribing physician is not necessarily present during the shift in which the medicine is administered. The preparation and documentation as well as the administration are usually done by educated and trained (nursing) staff. According to the commentary on §13 (2b) AMG, the essential characteristic is that the responsible person “must be able to assess the correctness of the preparation process and thus the quality and safety of the ready-to-administer preparation himself/herself, so that he/she can fulfill his/her professional responsibility, and he/she must personally administer the preparation” [33].

### Preparation of ready-to-administer medicinal products in hospital pharmacies

The preparation of ready-to-administer medicinal products for NICU patients by pharmaceutical staff in hospital pharmacies is especially indicated for complex and high-risk preparations, such as patient-specific parenteral nutrition admixtures. As a rule, the aseptic preparation of high-risk parenteral medicines should be carried out by pharmaceutical staff in dedicated clean rooms of the (hospital) pharmacy department [10], [34]. However, this general rule must not be detrimental to the individual patient, if less complex preparations are not available.

Depending on the staffing, premises and equipment, the pharmacy in charge can produce and supply "standardized ready-to-administer preparations” in advance [35]. This includes, for example, standardized ready-to-administer preparations for preterm newborns of different gestational ages to start immediate postnatal infusion therapy at any time.

### Aseptic handling of medicinal products by medical staff in clinical areas

This means that microbial contamination is prevented by appropriate hygienic conditions and measures during handling of parenteral medicines. The **aseptic non-touch technique (ANTT)** is a procedure in which the operator avoids touching key elements of a piece of equipment, such as the tip of a needle or the inside of a sterile dressing [36]. Aseptic handling by medical staff should be carried out at a “**KRINKO workplace**” (see below) and does not require the implementation of a clean room with various zones and a laminar air-flow bench.

### Specification of a workplace for the preparation of parenterally administered medicines on the ward according to the KRINKO recommendation (“KRINKO workplace”)

The preparation of all parenterally administered medicines (24/7) under clean-room conditions by pharmaceutical staff in a digitalized closed-loop procedure [35] fails due to its infeasibility and cost-benefit ratio. In a systematic review in 2015, Austin et al. pointed out that the investment and operating costs of such an "ideal solution" are immense and cannot be financed by income from ongoing business [12].

Focusing on infection prevention, the KRINKO has taken a clear position on this issue [9], [10]. It states:

*“The room in the ward where intravenous medicines are reconstituted according to the summary of product characteristics or where infusion solutions for preterm infants are prepared should meet the standard of a separate clean workroom in which all surfaces are suitable for wipe disinfection. The work surface on which patient-related reconstitution and preparation of parenteral medicines take place shall be wipe-disinfected before each operation. Windows and doors are to be kept closed during the relevant procedures. If there is a hand-washing area in this room, it should be far enough away from the work surface or screened off to prevent splashing water from contaminating materials or work surfaces.”*

Due to the risk of contamination posed by **sinks and taps**, the working group recommends removing all unnecessary sinks from clean workrooms used for the preparation of parenteral medicines [37]. Disinfectant dispensers for hand disinfection must be available and easily accessible.

According to the assessment of the WG and the results of the literature search, **the risk of airborne microbial contamination** during reconstitution of licensed medicinal products in a closed system with few handling operations is **lower than the risk of direct contamination by contact with contaminated hands or gloves** [38]. The risk of ingress of aerogenic pathogens is almost negligible during reconstitution [16]. As few people as possible should be in the room during reconstitution, and of course eating or drinking is not allowed.

### Laminar airflow (LAF) bench on the ward?

Workbenches with laminar air flow have been used in NICUs for many years in order to reduce the rate of microbial contamination during the preparation of parenteral medicines. In 2007, the KRINKO’s working group on neonatal intensive care recommended a “workroom for the aseptic preparation of medicinal products on wards with a workbench corresponding to the DIN 12980 Type H” [39]. Today, this concept has been superseded, since preparation of high-risk medicinal products takes place in pharmacies under the defined conditions of ‘Aseptic preparation and testing of ready-to-use parenteral medicines’ [34]. In principle, airborne contamination is much less important during the preparation of parenteral medicines than contamination by direct contact with contaminated hands, objects or surfaces [38]. 

In practice, inspections by hygienists indicate that working under the LAF bench on the ward may have no additional benefit in terms of microbial contamination risk, because

- the necessary framework conditions (installation in a suitable room, proper operating, monitoring by environmental controls) cannot be met,
- the rules of aseptic working under the LAF bench are not known and not reliably executed,
- the LAF bench does not function properly from a technical point of view (lack of maintenance, inspection or even lack of repair, e.g., due to delayed delivery or unavailability of spare parts).

Therefore, in the report on the joint meeting [2] and later also in the “Recommendation for the prevention of vascular catheter-associated infections in premature infants and neonates” [10] a clarification was made:

*“In order to ensure medication and patient safety, the reconstitution or preparation of parenteral medicines with a high risk of rapid multiplication of pathogens after contamination should preferably be carried out by the hospital pharmacy. In cases of low contamination risk the reconstitution or preparation can take place under proper hygienic conditions on the ward. An increased risk exists if the preparation steps are complex (e.g. manual mixing of numerous individual components for patient individually prescribed parenteral nutrition admixtures), the drug is a high-risk drug (such as lipid-containing infusion solutions) or if the preparation (such as hazardous medicinal products) is to be carried out under specific clean room conditions (e.g. clean rooms with negative pressure with safety workbenches) and requires specific education and comprehensive training of the ward staff. As a consequence, the preparation of patient-individual parenteral nutrition admixtures (macro- and micro-nutrients, electrolytes) should be exclusively performed in hospital pharmacies by experienced pharmaceutical staff.”*

### Urgent need for a complex infusion solution mixed for an individual patient

This is rare in NICUs. In most cases, standardized infusion solutions are on hand on the ward for transient use until the hospital pharmacy has prepared and supplied the complex mixed infusion prescribed for an individual patient. Nevertheless, every NICU should have ward-specific SOP regarding the immediate preparation of patient-specific parenteral nutrition admixtures. In such specific cases, it should be ensured that the immediate preparation of complex admixtures takes place on the ward under proper hygienic conditions and by qualified staff. The personnel must be educated and trained regarding the exceptional procedures.

### Clean rooms located on the ward

In some hospitals, the clean room for aseptic preparation of parenteral medicines is located in the patient care area (e.g., adjacent to the NICU). In this regard, the KRINKO makes the following recommendation [10]: 

*“A clean room for the preparation of high-risk medicinal products or infusion solutions can also be set up and operated on the ward. However, it must comply with all specifications for the preparation of parenteral medicines in a pharmacy in terms of design, function, workflow and staff qualifications (Cat. IV). The person responsible for the preparation of parenteral medicines in this clean room should preferably be the pharmacist in charge (Cat. IV).”*

Accordingly, it is irrelevant where such a clean room is located (in the hospital pharmacy department or on the ward adjacent to the NICU): the requirements for a proper workflow are identical [34].

### Personnel

When reconstitution/preparation takes place in clinical areas, the personnel involved in the preparation and administration of parenteral medicines must be educated (basic knowledge, specific knowledge) and trained (practice of the appropriate handling under supervision) [31]. In particular, education should provide knowledge and skills in calculating the required dose, hygiene and aseptic working techniques such as the ANTT. Training courses should be organized in cooperation with hospital pharmacists and hygienists. Participation in training courses must be documented and learning success should be proven in tests [4].

### Cleaning/disinfection

The cleaning/disinfection measures of a workplace intended for the reconstitution/preparation of parenteral medicines must be specified in a detailed sanitization plan. Type of cleaning/disinfection measures, timepoints and frequency are defined.

When entering the room, clean working clothes (not protective clothing) must be worn and hygienic hand disinfection must be carried out. Protective clothing, such as gowns, headgears, and face masks, should be worn when complex parenteral admixtures are prepared exceptionally in the clinical area. The cleaning staff must be educated in terms of clothing, cleaning, and disinfection regulations. Windows and doors should be closed during reconstitution. No eating or drinking is allowed in the room. As few people as possible should be in the room and no other activities should be carried out by other people at the same time. The work surfaces must be disinfected before starting work (wipe disinfection according to the sanitization plan). 

The recommendations of the KRINKO on *hand hygiene* [40] must be followed with regard to the performance of hand disinfection, time-points, the proper hand disinfectant solutions, and the use of non-sterile and sterile disposable gloves. The process of aseptic handling must always start with hygienic hand disinfection. This is mandatory, regardless of whether non-sterile or sterile disposable gloves are subsequently worn. If gloves are used, gloved hands should only be disinfected in special cases, e.g., in situations where frequent glove changes are necessary but not suitable or can lead to interruption of the workflow. Gloves should be disinfected when administration devices are (dis)connected to venous access devices [40]. Whenever possible, the reconstitution process of parenteral medicines should be completed and not interrupted by other activities or alarms.

*Multiple withdrawals of solutions from a single-dose container* intended to be administered to different patients are not allowed. Leftovers must be discarded immediately. Multiple withdrawals from a “single-use only” dosage form are only allowed during preparation in the hospital pharmacy [9], [41], [42]. Finished medicinal products that are authorized as *multi-dose containers* contain preservatives and show an appropriate declaration on the label (e.g., insulin, heparin). When multi-dose containers are used, the date and time of the first withdrawal must be noted. With regard to the type and duration of storage, the information in the summary of product characteristics must be strictly followed. Any handling of the medicinal products deviating from the summary of product characteristics may lead to serious consequences for patients and is the full responsibility of the attending physician. It must be assumed that a deviating procedure is not included in the patient consent given and can be interpreted in court as intentional bodily injury [43].

### One-hour rule 

*The one-hour period* represents the maximum time latency between the reconstitution of medicinal products for parenteral use and the start of i.v. administration: "*In this respect and after expert advice, the Court of Appeal states that infusion solutions should be prepared a maximum one hour before starting the application, in order to avoid growth of bacteria (after incidental contamination during handling) thus posing a risk of infection to the patient*” [44].

According to the European Resolution on good reconstitution practices for parenteral medicines in healthcare establishments CM/Res (2016)2, reconstitution should ideally take place as close as possible to the time of administration or use [31]. Preparation in advance is only allowed if ready-to-administer parenteral medicines are prepared under clean-room conditions in hospital pharmacies. In this case, pharmacists determine the shelf life of the parenteral medicines, declare an expiration date, and provide information on correct storage conditions and the limits of use on the label.

Even though the one-hour time limit was not established on the basis of scientific evidence, it is comprehensible and represents an important warning signal [16] for the clinical team. If the one-hour rule cannot be met for operational or organizational reasons (e.g., emergency medicines prepared in advance in the appropriate concentration in neonatology, anaesthesia, etc.), all team members should be aware of the increased medication risk [45]. Moreover, the liability of the physician in charge or organizational culpability of the responsible staff is avoided if all available options for providing ready-to-use parenteral medicines are considered. In this respect, hospital pharmacies play an essential role, either by purchasing marketed ready-to-administer parenteral medicines or by aseptic preparation of ready-to-administer parenteral medicines (including emergency medicines) according to the quality and safety assurance requirements.

## Reconstitution/preparation of parenteral medicines for NICU patients by the medical team

For infection prevention, the following workflow is suitable when reconstituting parenteral medicines and when connecting parenteral nutrition bags to administration devices (adapted from [16], [31], [41], [42], [36]):

- Hand disinfection when entering the room in which the "KRINKO workplace" is located [10]
- Collect the medicinal products used as starting material, sterile disposables (syringes, mini-spikes, cannulas) and other accessories (e.g., disinfectants, sterile compresses) required for reconstitution. Use a picking list if available.
- Visual inspection of the medicinal products used as starting material for discoloration, turbidity, and defects of the primary container.
- Wipe-disinfect the surface of the workplace with a proper disinfection solution and arrange the needed material suitable for a smooth workflow.
- Repetition of hygienic hand disinfection and – if non-sterile gloves are worn – disinfection of gloved hands. Each interruption of the workflow with handling outside the disinfected workplace requires new hygienic hand disinfection.
- Wipe-disinfection of glass ampoules with alcohol-impregnated wipe. When opening the ampoule, place a compress around its neck to minimize the risk of microbial contamination and injury.
- Remove the flip-off caps from vials of the medicinal product. Wipe- or spray-disinfect the rubber stoppers with alcoholic disinfectant solution and allow the disinfectant solution to dry (exposure time at least 15 seconds).
- Remove the sterile accessories from the packaging with peel-off technique.
- Do not place accessories on the work surface without protective caps.
- Touch plunger of syringes only at the plunger platform.
- Reconstitute (e.g., inject solvent, withdraw solution) without touching connection sites of devices (non-touch technique).
- For withdrawal volumes up to 50 ml, the injection of room air into rigid and semi-rigid containers is permitted to achieve pressure balancing.
- For withdrawal volumes >50 ml, spikes/dispensing pins should be used. This reduces the risk of bacterial contamination by automated pressure balancing via an integrated air channel equipped with a bacteria-retentive air filter (0.45 µm)).
- Withdrawal from multi-dose containers can be done with syringes and connected cannulas with a single-use technique or spikes/dispensing pin. Spikes must be equipped with a bacteria-retentive air filter and caps to reduce touch contamination of the critical Luer connection site. Tight-sealing caps improve working hygiene and potential leakages when closed. Each withdrawal is to be done with a new syringe. The connection site for the syringe can only be considered sterile directly after removal from the packaging. In case of repeated withdrawal, wipe-disinfection of the connection site with an alcohol-soaked wipe is recommended. After disconnection of the syringe, the cap is closed. Spikes should be used at the most for the declared usage period of the multiple-dose containers. In the case of accidental touch contamination, the spike and the multiple-dose vial must be discarded immediately.
- Check the ready-to-administer infusion solution for particles (visual inspection).
- The reconstituted medicinal products must be clearly labeled and stored as specified in the instructions for use.
- Parenteral medicines must not be transported in gown pockets or placed in the incubator.

## Administration of parenteral medicines to NICU patients

Peripheral and central venous catheters are used for intravenous access. Only single-lumen catheters with small diameters (e.g., peripherally inserted intravenous catheters (PIC) or umbilical vein catheters (UVC)) can be placed in the small vessels of the newborns, which hampers safe administration. In order to reduce volume load and infusion time, infusion solutions are often highly concentrated, which increases the risk of incompatibilities in the infusion device or catheter. In case of known or suspected incompatibilities, simultaneous infusion must be avoided, and for subsequent infusion, an intermediate rinsing step with as small volumes of compatible vehicle solution as possible is mandatory in order to avoid toxicity and loss of activity [46], [47].

The osmolarity of the infusion solution determines whether the application is possible via a peripheral or a central venous access. **In general, solutions with an osmolality >800 mosmol/l are administered using a central venous access to avoid peripheral thrombophlebitis and extravasation**. Furthermore, the pH value of the infusion solution is decisive for vein compatibility. Alkaline solutions in particular (e.g. aciclovir, ganciclovir, omeprazole) can damage the venous endothelium. If the solutions are not irritating to the veins, concentrated solutions can be administered as bolus injections into the catheter via the injection port of the administration device. 

The properties of the infusion equipment must also be considered. When small volumes of infusion solutions are administered via long infusion lines, their dead volume must be taken into account. To avoid underdosing, the infusion volume should be increased by a surplus quantity and volume of each ingredient (see section on Prescribing). Infusion filters are used in particular to eliminate particles, air, and endotoxins. In-line infusion filters should also have a small dead volume and be selected appropriately, i.e., 0.2 µm with/without a positively charged filter membrane for aqueous solutions and 1.2 µm filter membranes for lipid-containing emulsions (for further information on in-line filters see KRINKO Recommendation [10]).

There is an increased risk for incompatibilities in neonatal intensive care patients due to the use of single lumen catheters, high concentrations and the low flow rates of the infusion solutions. Incompatibility reactions are physicochemical reactions that can lead to decreased concentration of the active ingredient and to an increase in degradation products. In many cases, the incompatibility reaction leads to precipitation/precipitates, which cause an alarm at the infusion pump due to filter and catheter occlusion and may be visible in the infusion equipment. In preterm infants, there is a risk of granulomas in the terminal pulmonary circulation if infusion solutions contain insoluble particles. Therefore, all infusion accessories should always be inspected carefully. 

To avoid incompatibilities, **relevant information should be available and followed, e.g. in table format in the ward-specific manual on parenteral therapy**. Generally, information regarding compatibility is presented for pairs of two parenteral medicines by categorizing them as compatible, incompatible, contradictory information available, or no information available. Experimentally determined data often refer to adult doses and concentrations and are not readily transferable to the higher concentrations and longer contact times used in neonatal patients [46], [47]. In the case of consecutive application of incompatible infusions (e.g., due to strongly differing pH values), pre-rinsing and post-rinsing with vehicle solution (0.9% NaCl or 5% glucose solution) is mandatory [48]. The volume of the rinsing solution must be adapted to the dead volume of the infusion equipment and kept as low as possible. Only 0.9% NaCl or 5% glucose solution should be used as vehicle solution for infusion solutions, even if other solutions (e.g., Ringer's solution) are indicated as compatible in the summary of product characteristics. Infusion solutions with higher pH or bivalent cations (e.g., calcium, magnesium) bear a high risk of incompatibilities.

When preparing parenteral nutrition admixtures, incompatibility reactions (e.g., due to calcium ions, trace elements, different pH values, different densities) are relevant, but can be avoided by addition of the different components in the proper order. Fat emulsions (with or without vitamins) should be stored light protected. If possible, no other medicines (e.g. heparin) should be added to parenteral nutrition admixtures or administered simultaneously via the same infusion device or catheter lumen. If no compatibility information is available, a search request should be addressed to the pharmacy.

To avoid microbial contamination during application, **hygienic hand disinfection must be carried out before any manipulation of the infusion equipment and venous catheters** [40]. Connection sites must be disinfected with a suitable alcohol-impregnated wipe before each manipulation and, if not used, closed with sterile Luer lock cap. Connection sites must not be touched (according to the ANTT).

The correct setting of the infusion rate and infusion duration constitutes a critical factor to avoid dosing errors and variations in the applied volumes and doses. Dosing errors depend, among other things, on the characteristics of the infusion devices, especially the type of syringes and catheters used. By using syringes with smaller nominal volumes, the feed of the syringe plunger is more even, resulting in a minimization of unintended variations of the infusion rate during syringe replacement in the syringe pump. By closing all infusion lines during syringe replacement, most adverse events can be prevented or reduced [13].

### Risk identification

Risk identification is a prerequisite to assess and manage the risk related to infusion therapy in preterm/term newborns [31].

*Product and process-related risks are*

- Microbiological contamination (degree of complexity of reconstitution, nutritive properties of the medicinal product, storage period, infusion period)
- Incorrect composition (complex calculation, dose unit conversion errors, dilution of concentrates, multiple dilution steps, dissolution of powders, removal of partial quantities from ampoules or vials, incompatibility with vehicle solution, specific administration devices)
- Pharmacological activity of the active ingredient, narrow therapeutic range, steep dose/efficacy curve, e.g., potassium chloride, opiates, insulin, catecholamines)

Microbial contamination (unintended or accidental introduction of infectious material) of parenterally administered medicines is a major risk factor for nosocomial bloodstream infections (BSI). Vascular catheter-associated BSIs are among the most frequently documented nosocomial infections in very low birth-weight preterm neonates [14], [49]. When using proper hygiene measures in the right way, only a small number of blood stream infections (BSI) are caused by contaminated parenteral medicines [50], [51]. Reporting of nosocomial BSI in the NEO-KISS module is mandatory according to the German Infection Protection Act (*Infektionsschutzgesetz*) and the corresponding resolution of the Federal Joint Committee (G-BA) [52], [53].

The risk of microbial contamination and subsequent BSI increases with increasing growth promoting properties of parenterally administered medicines and with increasing growth rates of the contaminating bacteria [54], [55], [56], [57]. The risk is generally considered to be low if reconstitution of sterile finished medicinal products is performed with a low number of handling operations and immediately before application (less than 1 hour). The risk of harmful microbial contamination increases if more than five non-touch handling operations are performed, the reconstitution is performed as open-system procedure and if the product has growth-promoting properties (e.g. fat emulsions) [57]. 

The reconstitution of a parenteral medicine is more complex in neonatal and paediatric patients because the dose must be calculated for each patient individually in mg/kg body weight or mg/m^2^ body surface. The prescribed dose usually corresponds to a partial amount of a licensed medicinal product. 

However, multiple withdrawals from a single-dose container for different patients are not permitted; leftovers must be discarded immediately. Portioning of medicinal products from an ampoule or infusion bottle for several single doses or for several patients is only permitted to a pharmacist under validated conditions [9], [29]. When licensed multi-dose vials are used, the information in the summary of product characteristics regarding the type and duration of storage after first opening must be strictly followed. Although multiple dose vials contain preservatives and the medicinal products are less susceptible to microbial growth, multiple withdrawals bear a higher risk of microbial contamination. The proper use of devices (syringes, needles, spikes, pins) minimizes the risk of contamination (see chapter *Reconstitution/preparation of parenteral medicines for NICU patients by the medical team*). The use of spikes is advantageous when larger volumes are removed.

### Risk analysis

 The failure mode and effects analysis (FMEA) method is useful for risk analysis of the prescription, preparation and administration of intravenous medicines [5], [6], [58]. The highly structured FMEA allows the analysis of as many components and assemblies as possible for manifold processes. For each component, the failure modes and the resulting effects on the rest of the system are documented and calculated [59].

For each failure mode the following three items are assessed:

O = probability of Occurrence 

S = Severity: consequence for the system or the process when the error occurs.

D = probability of Detection

Next, a score (e.g., from 1 to 3) is assigned to each item of the failure mode, taking into account the local context and the process characteristics (see Table 2 (Tab. 2)). By multiplying the score of the likelihood of occurrence (O) by severity of the potential effect for the patient (S) by the chance of detecting the failure (D) before patient safety is affected (O x S x D), the risk priority number (RPN) is calculated. 

Hence, RPN values between 1 and 27 are to be expected, whereby a higher RPN indicates a higher risk for the patient. Failure modes with high RPNs require immediate attention and action to mitigate the risk (see Table 3 (Tab. 3)).

Comparison of the RPN before and after taking measures helps to evaluate whether the risk is reduced to an acceptable level. An exemplary FMEA for hand hygiene is shown in Table 4 (Tab. 4).

### Risk management

In this case, the aim of risk management is to reduce the risks associated with the prescription, reconstitution and administration of parenteral medicines in neonatal intensive care units. To minimize the risks, the range of products and presentations of parenteral medicines should be rationalized. If possible, the range of concentrations/strengths should be reduced to minimize the risk of selection errors [31]. Whenever possible, ready-to-administer or ready-to-use products should be provided by the pharmacy department. Preference should be given to vials rather than ampoules to reduce the risk of microbial contamination.

The risk of calculation errors can be reduced by electronic prescribing and automated generation of product-specific working instructions for reconstitution and administration. A second person should check the selection of starting materials to reduce the risk of incorrect composition. Printable or pre-printed labels promote clear and complete labeling.

Checklists and monitoring forms help to ensure monitoring and documentation throughout reconstitution and administration [31]. It is advisable to double-check calculations and other high-risk process steps according to the four-eye principle. Both team members (physician + nurse, nurse + nurse) check the identity and quantity of the starting material and sign the documentation form. Further quality controls such as physicochemical analyses are not required for the medicinal products prepared on the ward. The hygienic conditions at the workplace can be monitored by the hospital hygiene department using environmental monitoring methods (glove finger dabs, surface swab test, settle plates). A respectful and trusting relationship among all professionals involved creates an environment where a lack of self-confidence is accepted as a reason to seek help and obtain support [31]. At the same time, awareness and mutual control must be constantly present.

## Notes

### Competing interests

The authors declare that they have no competing interests.

### Acknowledgement

We are very grateful to Dr. Jörg Arand for the provision of an exemplary “Prescription and working instruction” resulting from a software program he developed and continuously updated.

We also express our sincere gratitude to Frank Erdnüß for his continuing and professional support to optimize the English wording of the manuscript.

### Authorship

IK und RG: Shared first authorship

### Annotation

The recommendation has been published in German with the following bibliography: 

Krämer I, Goelz R, Gille C, Härtel C, Müller R, Orlikowsky T, Piening B, Schubert S, Simon A, Wolf K, Rösner B, Exner M. Management der parenteralen Arzneimitteltherapie auf neonatologischen Intensivstationen – Positionspapier einer interdisziplinären Arbeitsgruppe. HygMed. 2022;47(6):118-28. 

## Figures and Tables

**Table 1:**
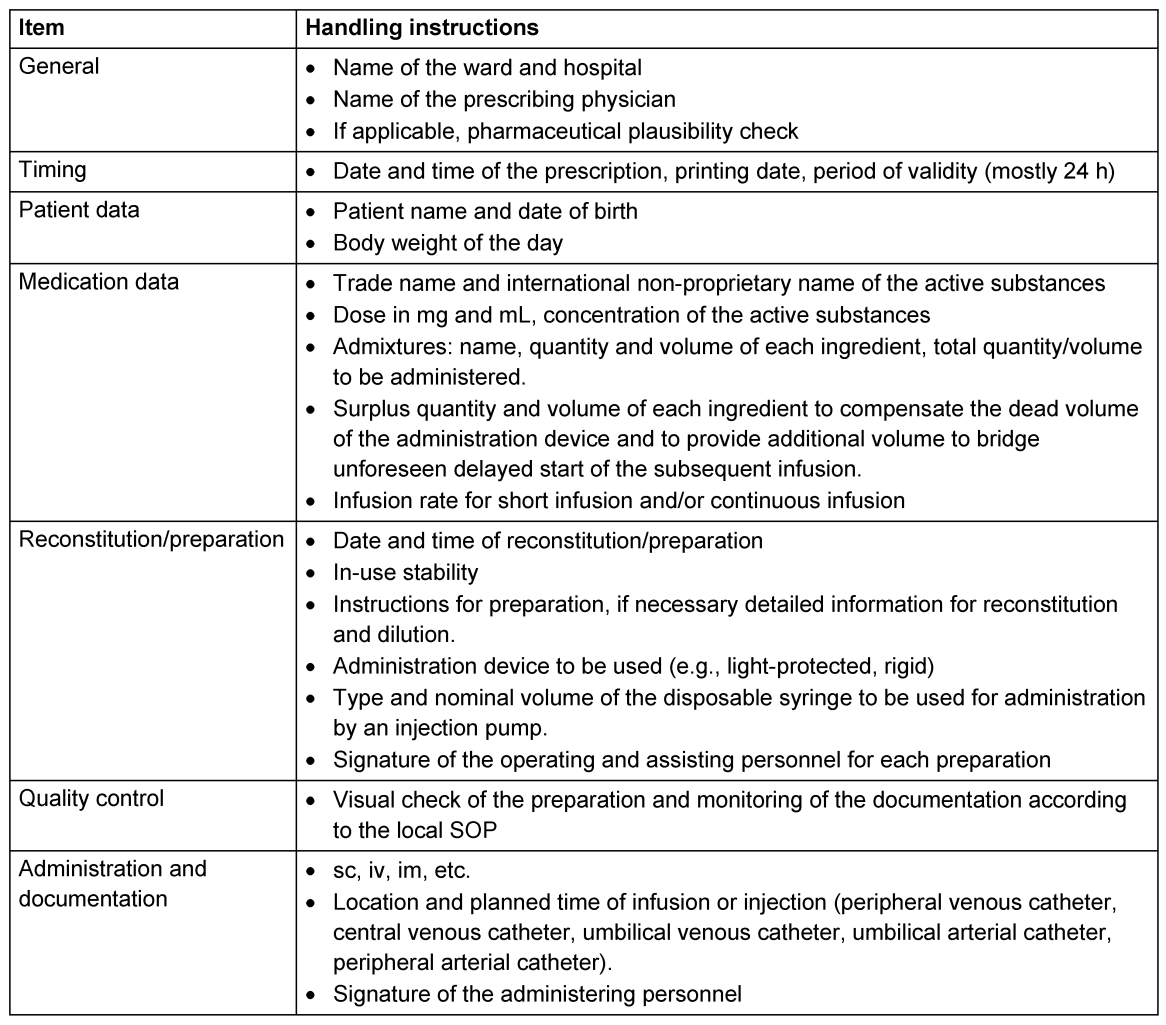
Relevant items of a prescription and handling instructions on the NICU

**Table 2:**
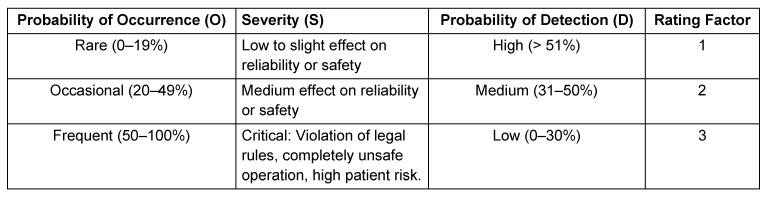
Example of a scoring scheme regarding probability of occurrence, severity, and probability of detection (Score 1–3)

**Table 3:**
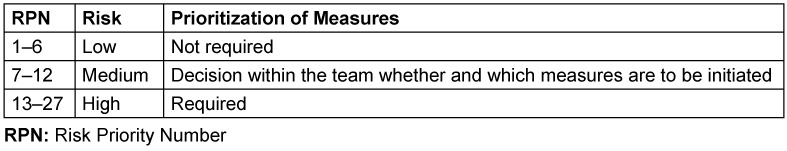
Prioritization of measures related to the risk priority number

**Table 4:**
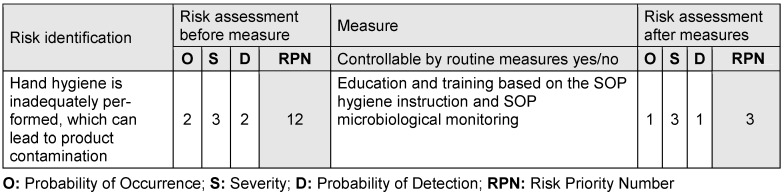
Example of a risk analysis before and after intervention
